# Contraceptive Prescribing and Dispensing After the Defense Health Agency’s Policy Change

**DOI:** 10.1001/jamanetworkopen.2025.39451

**Published:** 2025-10-27

**Authors:** Richard Juneau, Grishma KC, Alexander G. Rittel, Marissa Rittel, Jill Brown, Monica A. Lutgendorf, Krista B. Highland, Ryan C. Costantino, Laura E. Gressler

**Affiliations:** 1 The Henry M. Jackson Foundation for the Advancement of Military Medicine, Inc, Rockville, Maryland; 2 Department of Pharmaceutical Evaluation and Policy, University of Arkansas for Medical Sciences, Little Rock; 3 Enterprise Intelligence and Data Solutions, Program Management Office, Program Executive Office, Defense Healthcare Management Systems, San Antonio, Texas; 4 Department of Gynecologic Surgery and Obstetrics, Uniformed Services University of the Health Sciences, Bethesda, Maryland; 5 Department of Anesthesiology, Uniformed Services University of the Health Sciences, Bethesda, Maryland

## Abstract

**Importance:**

The Defense Health Agency Procedural Instruction (DHA-PI) 6200.02, introduced in May 2019, aimed to improve contraception access and knowledge among eligible beneficiaries. However, the policy’s association with contraception prescribing and dispensing practices has not been fully evaluated.

**Objective:**

To evaluate changes in contraceptive prescribing and dispensing practices associated with DHA-PI 6200.02, focusing on extended day supply of long-acting reversible contraception (LARC), short-acting reversible contraception (SARC), and permanent contraception.

**Design, Setting, and Participants:**

This retrospective cohort study was conducted utilizing interrupted time-series analysis to examine changes in contraception care practices before and after policy implementation. The study period was between January 1, 2016, and September 30, 2022. Participants were active-duty servicewomen (ADSW) aged 18 to 55 years, with an assigned sex of female in medical records who were enrolled in TRICARE.

**Exposure:**

The implementation of the DHA-PI 6200.02 policy in May 2019.

**Main Outcomes and Measures:**

The primary outcomes included rates of extended contraceptive supply and utilization of LARC, SARC, and permanent contraception. Five performance measures were employed to assess the associations between DHA-PI 6200.02 and changes in contraceptive supply. Segmented regression models were unadjusted and used to estimate level and trend changes in monthly MOPs.

**Results:**

Among 429 194 ADSWs (mean [SD] age 24.5 [7.0] years; 148 104 [34.5%] Army, 101 299 [32.6%] Navy, 115 288 [26.86%] Air Force, and 35 352 [8.24%] Marine Corps), the proportion of SARC users receiving extended supply increased from 1.1% prepolicy to 5.3% postpolicy. Extended SARC use increased from 16.3% (95% CI, 16.0%-16.7%) at baseline to 19.7% (95% CI, 17.9%-21.6%) at the end of follow-up, and dispensed extended SARC rose from 8.2% (95% CI, 7.9%-8.5%) to 9.3% (95% CI, 7.4%-11.1%), both reflecting significant post-policy upward trends. The overall proportion of ADSW with any extended contraceptive supply showed no meaningful change and declined significantly in trend. Prescriber provision of extended supply rose modestly without sustained trend change, and among contraceptive users extended supply increased but without significant policy-related effects.

**Conclusions and Relevance:**

In this cohort study of ADSWs, DHA-PI 6200.02 was associated with increased access to extended day supply for SARC but no corresponding increases were observed in overall extended contraceptive supply across all methods (SARC, LARC placement, and permanent contraception). These findings underscore challenges in policy uptake and highlight the need for enhanced efforts to ensure comprehensive implementation of DHA-PI 6200.02.

## Key Points

**Question:**

Was the release of Defense Health Agency Procedural Instruction (DHA-PI) 6200.02, which aimed to increase access to contraceptives among beneficiaries, associated with changes in contraceptive prescribing and dispensing patterns among active-duty servicewomen (ADSW)?

**Findings:**

In this cohort study of 429 194 ADSW, statistically significant increases were observed in extended-supply prescriptions of short-acting reversible contraceptives. However, the proportion of eligible ADSW receiving extended supply declined, and no statistically significant changes were seen in the number of prescribers providing extended supply.

**Meaning:**

These findings suggest partial uptake of the DHA-PI 6200.02 policy across the Military Health System, with opportunities for improvement with consistent implementation of extended contraceptive supply.

## Teaser

This cohort study evaluates changes in contraceptive prescribing and dispensing practices associated with Defense Health Agency (DHA) Procedural Instruction 6200.02, focusing on extended day supply of short-acting reversible contraception, long-acting reversible contraception, and permanent contraception.

## Introduction

Approximately 97% of active-duty servicewomen (ADSW) are of reproductive age (ie, 18-55 years) and face challenges related to accessing contraceptive care compared with the general population, particularly during deployment.^1,2^ In a 2020 survey, 22% of ADSW reported experiencing delay at least once in accessing birth control, and 6% reported being unable to obtain necessary birth control in the Military Health System (MHS).^3^ Another study found that one-third of surveyed ADSW lacked access to their desired contraceptive during deployment.^4^ Additionally, 59% did not discuss their contraceptive options with a practitioner beforehand, and 41% reported difficulty obtaining refills during their last deployment.^4^ Inadequate access to and supply of contraception throughout periods of military service and deployments can lead to unintended pregnancy, decreased readiness, mission degradation, and other adverse outcomes.^1,2,3,4,5^

Access to contraception has many health and operational benefits for ADSWs. The most recently reported unintended pregnancy rate among ADSW was estimated to be 72 per 1000 women, compared with 40.6 to 45 per 1000 civilian women in the same year.^6,7,8,9^ Unintended pregnancies can negatively impact military careers and potentially impede military unit readiness.^1,3,5^ Hormonal contraception can also help manage conditions such as acne, hirsutism, menstrual migraines, menstrual pain, and irregular menstruation.^10,11^ It also allows for menstrual suppression, which may benefit deployed service members.^10,11^ Therefore, promoting contraceptive care access can improve ADSW’s physical and mental well-being while facilitating military readiness and optimizing military resources.

To overcome barriers and improve access and education related to contraceptive care, numerous laws, policies, and clinical practice guidelines have been implemented by military health authorities.^12,13,14,15^ The Defense Health Agency Procedural Instruction (DHA-PI) 6200.02, issued in May 2019, aims to improve access to contraceptive care by addressing barriers and providing comprehensive counseling.^16^ eFigure 1 in Supplement 1 details the various laws, policies, and clinical practice guidelines before and after the release of DHA-PI 6200.02 that support its aims. DHA-PI 6200.02 was designed to increase access to contraception among ADSWs by (1) ensuring the availability of all US Food and Drug Administration–approved contraceptive methods at military treatment facilities, (2) authorizing up to a 12-month supply of short-acting reversible contraceptives (SARCs) from military pharmacies, (3) enabling the use of long-acting reversible contraception (LARC) when appropriate, and (4) mandating practitioner education to ensure comprehensive contraceptive counseling and documentation across the MHS. These provisions were intended to reduce logistical barriers (eg, difficulty obtaining refills while deployed), expand method choice, and promote consistency in care delivery.^17,18,19^

DHA-PI 6200.02 ensures that ADSW receive an adequate supply of SARCs to support deployment readiness and allows for dispensing up to a 12-month supply through military pharmacies.^5^ It also includes provisions for menstrual suppression.^5^ This aims to reduce clinic visits and unintended pregnancies, enhance adherence, and contribute to mission readiness.^20,21^ The policy also mandates timely access to contraception within 24 hours for ADSW before deployment, promotes counseling on LARC, and addresses storage challenges with SARC.^15,22^ Additionally, it requires annual evidence-based family planning education, covering contraceptive options, emergency contraception, menstrual suppression, and chronic condition management.^16^

While the DHA-PI 6200.02 attempts to improve contraceptive care access, education, and counseling, its implementation and associated outcomes have not been evaluated. This study leveraged a pragmatic, retrospective design^23,24^ to evaluate the implementation of DHA-PI 6200.02 using implementation science constructs such as adoption and fidelity, with a focus on extended contraception supply and related prescribing and dispensing practices within the MHS.

## Methods

### Data Source and Setting

This retrospective observational cohort study included ADSW enrolled in TRICARE between January 1, 2016, and September 30, 2022. Data were obtained via the MHS Information Platform, which includes enrollment, medication, and encounter data for both direct and purchased care encounters. Direct care refers to services provided within military treatment facilities (MTFs), while purchased care refers to services rendered in the civilian sector and reimbursed by TRICARE. The Defense Eligibility Enrollment Registration System was utilized to identify ADSW who met inclusion criteria. The Pharmacy Data Warehouse provided fill-level data on dispensed medications to TRICARE beneficiaries, including most recent fill date and days’ supply. Inpatient and outpatient encounter data came from the Comprehensive Ambulatory Professional Encounter Record and TRICARE encounter data (institutional and noninstitutional). These sources enabled comparisons between direct care (MTFs) and purchased care (civilian health care networks). Demographic and service characteristics were extracted during the first month of inclusion. Race and ethnicity were self-reported and ascertained through the Defense Eligibility Enrollment Registration System enrollment records; data on race and ethnicity are included here because xx. This study followed the Strengthening the Reporting of Observational Studies in Epidemiology (STROBE) reporting guideline.^25^ This study was reviewed and deemed exempt by the DHA Institutional Review Board, with a waiver of informed consent granted due to the use of deidentified secondary data.

### Study Cohort

Eligible records included ADSW aged 18 to 55 years with sex listed as female in medical records who were enrolled in TRICARE for any length of time during the study period. Continuous enrollment was not required. Individuals were counted in the numerator and denominator if enrolled in the month a contraceptive was dispensed or a procedure performed, and in each subsequent month until disenrollment, pregnancy, or other exclusions. ADSW affiliated with the Coast Guard or with missing key data were excluded. Pregnancy periods, identified using *International Statistical Classification of Diseases and Related Health Problems, Tenth Revision (ICD-10) *diagnosis codes and ending at delivery (*Current Procedural Terminology* procedure codes), termination (*ICD-10*), or restart and/or initiation of a SARC, LARC placement, or permanent sterilization, were excluded to avoid misclassifying planned discontinuation as lack of access or policy noncompliance. Additionally, the following exclusion criteria were applied. The first was no receipt of contraception (SARC, LARC, or permanent contraception^26^) or used diaphragms, external or internal condoms, spermicides, or natural family planning (withdrawal or fertility-based awareness methods) as their only form of contraception. The second criterion was receipt of emergency contraception only, defined as (1) 1 or more ulipristal acetate or levonorgestrel prescription or (2) a single package (prescription quantity <30 pills) of a combined hormonal contraception identified as part of the Yuzpe method with a diagnosis of *ICD-10* code Z30.012 (encounter for emergency contraception).

### Outcome

As previously described, DHA-PI 6200.02 was designed to improve contraceptive access by addressing known barriers and supporting comprehensive counseling; this study focused specifically on the implementation of the policy’s provisions related to extended contraceptive supply. The primary outcome was the monthly rates of extended contraceptive supply defined as (1) LARC placement or SARC prescriptions with greater than or equal to 168 days’ supply or (2) permanent contraception without reversal. Secondary outcomes included (1) LARC placement or SARC prescriptions with greater than or equal to 364 days to address policy fidelity and (2) percentage of prescribers who provided extended forms of contraceptive supply to assess market penetration. SARC supply was considered to start on the first day of the dispensing month. Supply duration was rounded to indicate months of contraception supply (eg, 84 pills dispensed January 15 equaled 3 months: January [index month] to March). LARC supply was based on type and formulation.

Because claims data reflect utilization and not intent or preference, our measures represent proxies for access and continuity, not direct measures of contraceptive need or decision making. Four subgroups were created on the basis of contraception receipt: SARC, LARC, permanent contraception, and no contraception. The identification and quantification of the included contraceptives are summarized in eMethods 1 in Supplement 1. ADSW could be in more than 1 subgroup during the study period based on the contraception received with the exception of the no contraception group. The no contraception group included ADSW who were not using any contraception (SARC, LARC placement, or permanent contraception) at any point during the study period.

### Covariates and Measures of Performance

Product-specific and procedure-specific lookback periods of up to 10 years were used to identify contraceptive use that extended into the study period. This study was designed to evaluate the implementation of DHA-PI 6200.02 using constructs from implementation science. Specifically, we assessed (1) adoption, or the initial uptake of extended contraceptive supply practices among eligible prescribers and patients; (2) fidelity, or the extent to which contraception was provided in accordance with the policy’s intended duration (eg, 12-month SARC supply), (3);market penetration, or the degree to which extended contraceptive supply became embedded across the MHS prescriber base; and (4) sustainability, or the maintenance of these practices over time.

To operationalize these constructs, we defined 5 measures of performance (MOPs). The first, MOP 1 (adoption), was defined as the monthly percentage of eligible ADSW with extended SARC supply (≥168 days) among those with an active SARC prescription. The second, MOP 2 (fidelity), was defined as the monthly percentage of SARC prescriptions dispensed for greater than or equal to 168 days’ supply. The third, MOP 3 (adoption), was defined as the monthly percentage of eligible ADSW with any extended contraceptive supply (SARC, LARC placement, or permanent contraception). The fourth, MOP 4 (market penetration), was defined as the monthly percentage of practitioners who provided any form of extended contraceptive supply (≥168 days SARC, LARC placement, or permanent contraception procedure). The fifth, MOP 5 (adoption), was defined as the monthly percentage of ADSW receiving any contraceptive method who received extended contraceptive supply. All MOP numerator and denominator definitions are detailed in eMethods 2 in Supplement 1.

### Statistical Analysis

Cohort demographics and service characteristics were reported according to first-month group eligibility. Interrupted time-series (ITS) analysis using segmented regression, conducted in R statistical software version 4.0.2 (R Project for Statistical Computing), was used to assess changes in MOPs associated with the policy. ITS is a robust quasi-experimental approach that can assess the longitudinal changes of the DHA-PI 6200.02 while distinguishing associations attributable to DHA-PI 6200.02 from underlying trends. For this study, the ITS analysis included data points from January 1, 2016, to January 31, 2019, as the preintervention period; and from September 1, 2019, to September 30, 2022, as the postintervention period. The implementation month (May 2019) and a 3-month washout period before and after implementation (February 1 to May 13, 2019, and May 14 to August 31, 2019) were excluded to account for potential delays in observed changes following the policy rollout.

Segmented regression models were unadjusted and used to estimate level and trend changes in monthly MOPs. Subgroup analyses compared changes between direct and purchased care settings. Two-sided *P* < .05 was considered significant.

## Results

**Figure 1.**
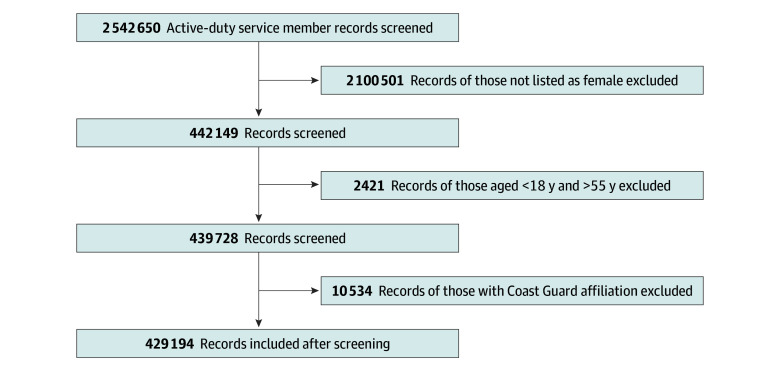
Study Flow Diagram Showing Identification, Exclusions, and Final Analytic Sample for the Analysis

A total of 429 194 ADSW (mean [SD] age, 24.5 [6.97] years) met inclusion criteria, regardless of contraceptive use (Figure 1). Of these, 148 104 (34.5%) served in the Army, 101 299 (32.6%) in the Navy, 115 288 (26.86%) in the Air Force, and 35 352 (8.24%) in the Marine Corps. Characteristics of ADSW at the time of initiating each form of contraception are presented in Table 1. Because individuals could initiate more than one method, counts are not mutually exclusive. Across the study period, 190 291 ADSW received SARC, 127 388 underwent LARC placement, and 14 168 received permanent contraception. Among those with SARC or LARC use, the majority were aged 18 to 24 years (112 036 [58.9%] for SARC; 73 804 [57.9%] for LARC), whereas permanent contraception was most common among ADSW aged 25 to 34 years (6760 [47.7%]). In the no-contraception group (n = 132 402), most were also aged 18 to 24 years (85 664 [64.7%]). During the study period, there were 1 663 613 direct care prescriptions and 305 233 purchased care prescriptions, provided by 30 734 direct care practitioners and 51 826 purchased care practitioners.

**Table 1.** Demographics and Service-Related Characteristics of Included ADSW in the First Month They Met a Subgroup Criteria^a^

Characteristics	ADSW, No. (%)			
	SARC (n = 190 291)	LARC (n = 127 388)	Permanent contraception (n = 14 168)	No contraception-overall (n = 132 402)^b^
Age group, y				
18-24	112 036 (58.9)	73 804 (57.9)	1025 (7.2)	85 664 (64.7)
25-34	60 953 (32.0)	41 703 (32.7)	6760 (47.7)	29 081 (22.0)
35-44	15 851 (8.3)	10 807 (8.5)	5777 (40.8)	12 253 (9.3)
≥45	1451 (0.8)	1074 (0.8)	606 (4.3)	5404 (4.1)
Service				
Army	61 833 (32.5)	36 536 (28.7)	5170 (38.5)	52 212 (39.4)
Air Force and/or Space Force	56 113 (29.5)	34 538 (27.1)	4931 (34.8)	30 863 (23.3)
Navy	36 683 (19.3)	30 606 (24.0)	2614 (18.5)	32 005 (24.2)
Navy Afloat	17 568 (9.2)	12 030 (9.4)	660 (4.7)	5081 (3.8)
Marine Corps	16 773 (8.8)	12 839 (10.1)	559 (4.0)	10 283 (7.8)
Other^c^	1321 (0.7)	839 (0.7)	234 (1.7)	1958 (1.5)
Rank				
Junior Enlisted (E1-E4)	117 205 (61.6)	75 590 (59.3)	2407 (17.0)	93 426 (70.6)
Junior NCO (E5-E6)	34 737 (18.3)	24 741 (19.4)	6311 (44.5)	12 817 (9.7)
Enlisted, Senior NCO (E7-E9)	7134 (3.8)	4955(3.9)	2920 (20.6)	5268 (8.8)
Junior Officer (O1-O3)	21 178 (11.1)	14 725 (11.6)	1033 (7.3)	11 627 (4.0)
Senior Officer (O4-O10)	5794 (3.0)	4307 (3.4)	1244 (8.8)	5798 (4.4)
Unknown	3465 (1.8)	2518 (2.0)	(<0.05)	2879 (2.2)
Warrant Officer (W1-W5)	778 (0.4)	552(0.4)	250 (1.8)	587 (0.4)
Race and ethnicity				
Asian (Non-Hispanic)	7372 (3.9)	4766 (3.7)	443 (3.1)	5919 (4.5)
Black (Non-Hispanic)	28 219 (14.8)	15 072 (11.8)	2281 (16.1)	20 280 (15.3)
Hispanic	23 505 (12.4)	17 142 (13.5)	1452 (10.3)	16 335 (12.3)
Non-Hispanic White	46 440 (24.4)	35 261 (27.7)	3608 (25.5)	30 466 (23.0)
Unknown	79 551 (41.8)	51 243 (40.2)	5859 (41.4)	56 337 (42.5)
Other^d^	5204 (2.7)	3904 (3.1)	525 (3.7)	3065 (2.3)

Abbreviations: ADSW, active-duty servicewomen; LARC, long-acting reversible contraceptive; NCO, noncommissioned officer; SARC, short-acting reversible contraceptive.

^a^ This table only represents ADSW in different subgroups during the first month of the study period. These subgroups are not exclusive throughout the study period; ADSW can be in more than 1 subgroup during the study period.

^b^ The no contraception subgroup included ADSW in the enrollment files who did not receive SARC, LARC, or permanent contraception during the entire study period.

^c^ The category other encompasses the following: The Commissioned Corps of the National Oceanic and Atmospheric Administration, Commissioned Corps of the Public Health Service, Foreign Air Force, Foreign Army, Foreign Marine Corps, Foreign Navy, and the Office of the Secretary of Defense.

^d^ The category other, as self-reported and recorded in enrollment records, is selected when beneficiaries do not feel represented by the listed categories or identify with multiple categories.

### MOP 1: Monthly Percentage of Eligible ADSW With Extended SARC Days Supply

**Figure 2.**
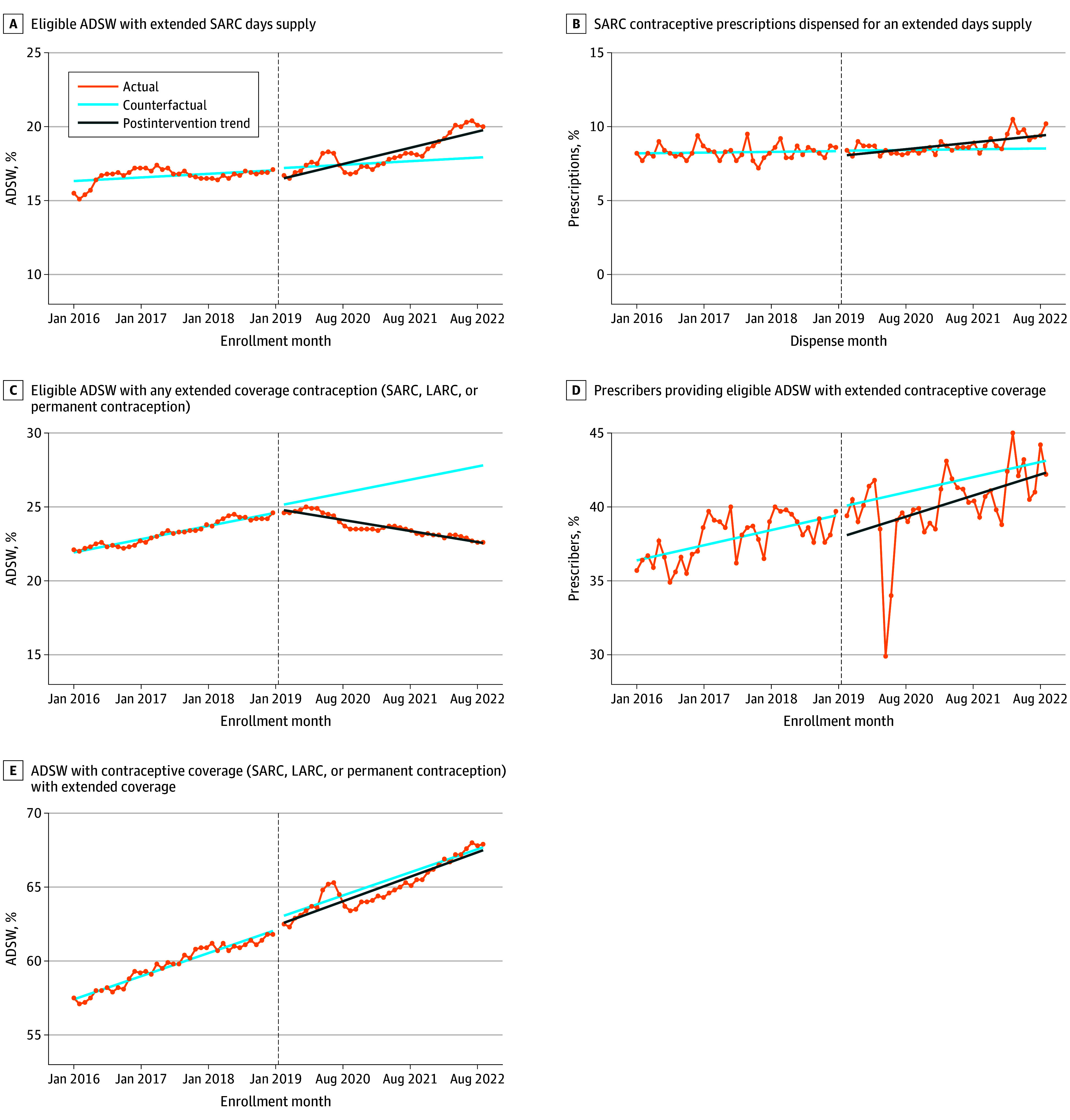
Interrupted Time-Series Analysis for Each Measure of Performance (MOP) Graphs show monthly percentage of eligible active-duty servicewomen (ADSW) with extended short-acting reversible contraception (SARC) days supply (A; MOP 1), monthly percentage of SARC prescriptions dispensed for an extended days supply (B; MOP 2), monthly percentage of eligible ADSW with any extended coverage contraception (SARC, long-acting reversible contraception [LARC], or permanent contraception) (C; MOP3), monthly percentage of prescribers providing eligible ADSW with extended contraceptive coverage (D; MOP4), and monthly percentage of ADSW with contraceptive coverage (SARC, LARC, or permanent contraception) with extended coverage (E; MOP5).

In January 2016, 16.3% (95% CI, 16.0% to 16.7%) of eligible ADSW had an active extended SARC prescription, increasing to 19.7% (95% CI, 17.9% to 21.6%) by the end of follow-up (Figure 2A). Although a positive trend was observed prior to DHA-PI 6200.02, a decrease of 0.77 percentage points (95% CI, –1.34 to –0.20 percentage points; *P* = .01) occurred immediately after the policy implementation, followed by a significant increase after intervention of 0.07 percentage points per month (95% CI, 0.05 to 0.09 percentage points per month; *P* < .001). In subgroup analyses, direct care settings showed a positive trend (coefficient, 0.09), while purchased care showed a negative trend (coefficient, –0.19) (eFigure 2 in Supplement 1).

### MOP 2: Monthly Percentage of SARC Prescriptions Dispensed for an Extended Days Supply

Extended SARC prescriptions dispensed increased from 8.2% (95% CI, 7.9% to 8.5%) to 9.3% (95% CI, 7.4% to 11.1%) during the postintervention study period (Figure 2B). The level change after the intervention was –0.34 percentage points (95% CI, –0.83 to 0.15 percentage points; *P* = .18), and a significant postintervention increase of 0.03 percentage points per month was observed (95% CI, 0.01 to 0.05 percentage points per month; *P* = .001). Subgroup analyses showed an increased trend in direct care (coefficient, 0.05), compared with a decreased trend in purchased care (coefficient, –0.12) (eFigure 2 in Supplement 1).

### MOP 3: Monthly Percentage of Eligible ADSW With Any Extended Supply Contraception

At baseline, 21.8% (95% CI, 21.7%-22.0%) of ADSW had extended supply, increasing to 22.1% (95% CI, 21.1% to 23.1%) postintervention (Figure 2C). A preintervention upward trend was observed (coefficient, 0.07; 95% CI, 0.06 to 0.08; *P* < .001), followed by a change of –0.25 percentage points (95% CI, –0.51 to 0.01 percentage points; *P* = .06) that was not statistically significant and a significant postintervention decrease (coefficient, –0.14; 95% CI, –0.16 to –0.12; *P* < .001). This decline occurred in both direct care (–0.14) and purchased care (–0.02) (eFigure 2 in Supplement 1). Analysis by method showed LARC placement followed a parabolic decline, while SARC and permanent contraception remained steady (eFigures 3-5 in Supplement 1).

### MOP 4: Monthly Percentage of Prescribers Providing Eligible ADSW With Extended Contraceptive Supply

At baseline 36.3% (95% CI, 35.1%-37.5%) of prescribers provided any extended contraceptive supply, increasing to 41.9% (95% CI, 35.8 to 47.9) by the end of the study period (Figure 2D). A small but significant decrease of –2.03 percentage points was observed immediately following the policy (95% CI, –3.95 to –0.11 percentage points; *P* = .04), with no significant postintervention slope change (coefficient, 0.03; 95% CI, –0.05 to 0.11; *P* = .41). Subgroup trends in direct (coefficient, 0.02) and purchased (coefficient, 0.05) care settings remained stable.

### MOP 5: Monthly Percentage of ADSW With Contraceptive Supply With Extended Supply Contraception

Among ADSW using contraception, extended supply increased from a baseline of 57.3% (95% CI, 57.0% to 57.6%) to 67.7% (95% CI, 66.1% to 69.2%) in the postintervention study period (Figure 2E). The level change at policy implementation was –0.48 percentage points (95% CI, –1.02 to 0.06 percentage points; *P* = .08), and no significant postintervention trend change was detected (coefficient, 0.008; 95% CI, –0.01 to 0.03; *P* = .48). No statistically significant trend was observed among direct (coefficient, −0.004) and purchased (coefficient, 0.005) care groups.

All outcome estimates are based on unadjusted segmented regression models using monthly aggregated rates. Full ITS model coefficients and SEs are reported in Table 2.

**Table 2.** Interrupted Time-Series Model Estimates Across MOP

ITS parameters	MOP 1^a^		MOP 2^b^		MOP 3^c^		MOP 4^d^		MOP5^e^	
	Estimate (SE)	*P* value	Estimate (SE)	*P* value	Estimate (SE)	*P* value	Estimate (SE)	*P* value	Estimate (SE)	*P* value
Baseline	16.30 (0.18)	<.001	8.19 (0.16)	<.001	21.84 (0.08)	<.001	36.30 (0.61)	<.001	57.30 (0.17)	<.001
Slope	0.02 (0.01)	.02	0.004 (0.01)	.57	0.07 (0.004)	<.001	0.08 (0.03)	<.004	0.13 (0.008)	<.001
Level change at intervention	−0.77 (0.29)	.01	−0.34 (0.25)	.18	−0.25 (0.13)	.06	−2.03 (0.98)	.04	−0.48 (0.27)	.08
Postintervention slope change	0.07 (0.01)	<.001	0.03 (0.01)	<.002	−0.14 (0.01)	<.001	0.03 (0.04)	.41	0.008 (0.01)	.48

Abbreviations: ADSW, active-duty servicewomen; ITS, interrupted time series; LARC, long-acting reversible contraception; MOP, measure of performance; SARC, short-acting reversible contraception.

^a^ MOP 1 is the monthly percentage of eligible ADSW with extended SARC days supply.

^b^ MOP 2 is the monthly percentage of SARC prescriptions dispensed for an extended days supply.

^c^ MOP 3 is the monthly percentage of eligible ADSW with any extended supply contraception (SARC, LARC placement, or permanent contraception).

^d^ MOP 4 is the monthly percentage of prescribers providing eligible ADSW with extended contraceptive supply.

^e^ MOP 5 is the monthly percentage of ADSW with contraceptive supply with extended supply contraception (SARC, LARC placement, or permanent contraception).

## Discussion

In this cohort study, the percentage of eligible ADSW receiving an extended supply of SARC (MOP 1) and the percentage of extended supply SARC prescriptions dispensed (MOP 2) significantly increased following the implementation of DHA-PI 6200.02. Although sustained trends were observed for SARCs, only positive but not statistically significant trends were seen for extended supply of SARC, LARC placement, or permanent contraception among contraception users (MOP 5) and practitioner uptake of extended supply contraceptive (MOP 4). However, MOP 3, which captured all eligible ADSW with extended supply, showed a significant decrease after DHA-PI 6200.02, indicating a lack of sustainability. Given the positive postintervention slopes in MOP 1 and MOP 5, the negative trend in MOP 3 could be due to declining number of ADSW seeking contraception.

Study findings suggest limited adoption, with all 5 MOPs seeing a decrease in treatment-level change at intervention, ranging from −0.25 to −2.03 percentage points (Table 2). Low fidelity was observed, as most ADSW who received an extended supply of a SARC received a 6-month supply rather than a 12-month supply. The postintervention slope for the number of practitioners offering extended supply (MOP 4) neither reached the counterfactual rate nor achieved statistical significance, suggesting limited market penetration and integration into practice across practitioners. Despite statistically significant changes, the absolute gains in SARC-related MOPs were modest (2%-5%) and below implementation science benchmark of 9%.^27,28^

Moreover, the observed increases appear to have occurred primarily in direct care settings. In contrast, purchased care, where TRICARE reimbursement and physician practices are more variable, showed smaller or negative changes. These differences may reflect systemic constraints, such as TRICARE’s historical cap on SARC supplies exceeding 3 months in purchased care, contextual factors like practitioner availability, deployment or leave status, and geographic access to MTFs. Although direct and purchased care represent the same beneficiary population, individuals may move between settings based on duty station or life circumstances, introducing additional complexity.

These findings show that while DHA-PI 6200.02 may be associated with increases in the dispensing of SARC extended day supplies, such changes warrant careful interpretation. SARCs offer flexibility and may align with user preferences for perceived control or fewer adverse effects. However, they require consistent access for refills, which can be interrupted by deployments or relocations. Some ADSW may prefer LARCs or permanent methods but opt for SARCs due to limited practitioner access, medical benefit reimbursement challenges, or delayed appointments. These utilization patterns highlight the importance of aligning contraceptive counseling with both medical eligibility and service member preference, within operational constraints.

These results align with the 2018 Health Related Behaviors Survey^29^ and the 2020 Women’s Health Reproductive Survey,^30^ which identified persistent barriers to widespread use of highly effective contraceptive methods among female service members. The Health Related Behaviors Survey reported lower-than-expected use of highly effective contraceptive methods and an overall usage rate below the Healthy People 2020 target.^31^ Similarly, the Women’s Health Reproductive Survey reported that only 28% of ADSW used highly effective methods such as LARC placement or permanent contraception.^30^

Several factors may explain the lack of significant change observed. Systemic logistical barriers within the MHS—such as inventory management processes and contraceptive availability—may hinder timely access. Variability in practitioner adherence to new policies and a potential lack of necessary training or resources may have contributed to inconsistent policy implementation. Additionally, the frequent relocations and deployments of ADSW and their clinicians complicate sustained access to contraceptive care, disrupting the continuity of care.^32^ The temporal overlap of DHA-PI 6200.02 implementation and the COVID-19 pandemic, which led to a sharp decline in contraceptive care appointments with visit volumes remaining below prepandemic levels throughout 2020,^33^ further complicates interpretation. This trend supports the notable decline in LARC placement and the slight decrease in permanent contraception (eFigures 3 and 5 in Supplement 1), both of which require in-person appointments, unlike SARC, which can be obtained virtually. Our data reflect this decrease; in particular, MOP 4 exhibited a sharp decrease in the number of practitioners prescribing extended supplies in 2020. While the numbers later improved, the trend in 2020 declined—contrasting with the upward trend seen before the pandemic, and making it difficult to isolate policy-related changes from pandemic-driven trends. Moreover, a cross-sectional study utilizing claims data found that the volume of intrauterine device services and prescriptions for the pill, patch, and ring remained below 2019 levels through end of 2022.^34^ These disruptions may have hindered the implementation of DHA-PI 6200.02.

A broader issue is the recurring gap between policy and practice seen in other military policies.^35,36^ Effective adoption requires communication, training, and support for both practitioners and ADSW. Misconceptions and information gaps, especially around LARC methods, may shape practitioner behavior and service member choices.^37,38^ Passing a policy is only the first step; without leadership accountability in its implementation, meaningful change is often limited.^39^ While clinical guidelines often shape care delivery, the extent to which Department of Defense or DHA policies exert similar influence remains uncertain. To strengthen uptake of DHA-PI 6200.02, the organization could apply implementation science tools such as the AACTT (action, actor, context, target, time) framework and Behavior Change Wheel to define actionable behaviors, identify key actors, and ensure sustainable implementation.^40,41^

### Strengths and Limitations

A key strength of this study is its large sample size and use of a rigorous ITS analysis, allowing for the detection of both immediate and gradual changes in MOPs. The multifaceted assessment of MOPs provides a detailed understanding of the policy’s impact.

However, several limitations should be noted. Before the implementation of DHA-PI 6200.02 in 2017, other policies related to contraception access, including the 2013 and 2016 Selected Practice Recommendations and DHA-Interim Procedures Memorandum, were introduced, and could have contributed to the observed changes in contraceptive prescribing and dispensing.^15,22^ Also, the study cannot determine whether the downward trend observed in MOP 3 (eFigures 3-5 in Supplement 1) was due to DHA-PI 6200.02, system-related barriers, or ADSW preferences.

The policy’s implementation overlapped with the COVID-19 pandemic, which introduced substantial disruptions to in-person health care delivery. These disruptions may have contributed to the decline in LARC and permanent contraception uptake observed in the study, confounding our ability to disentangle the effects of the policy from pandemic-related service limitations. The observed decline in practitioner-level MOPs (MOP 5) in 2020 likely reflects COVID-19–related staffing challenges and appointment cancellations, which affected both physicians availability and patient access. 

The cohort was limited to eligible ADSW receiving contraception through TRICARE, potentially underestimating the overall use of contraception among those not reimbursed or covered by TRICARE. We might be overestimating the cohort by including ADSW up to age 55 years; however, we wanted to also focus on contraceptive use for nonreproductive reasons such as use for menstrual suppression or perimenopausal symptoms. Additionally, TRICARE’s restriction on dispensing more than a 3-month supply in non-MTF pharmacies may have affected access and outcomes in purchased care, contributing to the observed differences between direct and purchased care settings. Although both settings serve the same beneficiary population, service members may shift between them due to duty station changes, deployment or leave status, or regional access to civilian practitionerss. These factors, along with variability in physician practices and formulary access, likely influence contraceptive supply patterns and implementation success.

Cadets were excluded because of the different context of their service compared with other branches, which might affect generalizability. Other uniformed service members, like United States Public Health Service and National Oceanic and Atmospheric Administration members, were not specifically focused on in this study but they may have been captured in the other service category. Moreover, increased operational tempo, including deployments, frequent relocations, and predeployment health assessments, may have shaped patterns in prescribing behavior, yet we were unable to capture or quantify these dynamics directly.

This study also relied on administrative claims data, which are subject to coding limitations, potential misclassification, and a lack of clinical nuance. Our method for calculating days’ supply of oral contraceptives may not fully reflect real-world usage patterns, particularly in cases of menstrual suppression or intermittent use. Moreover, claims data do not capture reasons behind contraceptive selection, which is influenced by individual preferences, practitioner counseling, perceptions of adverse effects, and cultural or structural stigma—factors that could not be directly assessed here.

Finally, although the large dataset enhances generalizability and statistical power, residual confounding and potential type I error remain possible. Although ITS is a robust quasiexperimental method, unmeasured time-varying confounders may still bias estimates. These considerations underscore the need for cautious interpretation of observed associations and reinforce the importance of triangulating findings with qualitative or prospective studies. Future studies should investigate barriers to contraception policy adherence, focus on specific types of contraceptive users, particularly those seeking care outside of TRICARE, and assess how educational interventions for both practitioners and service members affect policy outcomes.

## Conclusions

While this cohort study found DHA-PI 6200.02 was associated with statistically significant changes in extended SARC supply, broader policy uptake and sustainability across all contraceptive types remained limited. Key implementation outcomes, such as adoption, fidelity, and market penetration, remained low, although improvements related to SARC supply appeared to be sustained over time. These findings highlight the complexity of policy implementation within the MHS and suggest a need for greater practitioner support, policy assessment, and use of implementation science frameworks to drive meaningful change. Ongoing efforts will be essential to ensure that ADSW receive consistent, high-quality contraceptive care that supports both their health and operational readiness.

## Notes

Supplement 1. eFigure 1. Department of Defense (DOD) contraceptive policy timeline eMethods 1. Identification and quantification of contraceptive methods eMethods 2. Measures of Performance (MOPs) Numerator and Denominator Definitions eFigure 2. Direct vs purchased care interrupted time series analysis eFigure 3. Monthly percentage of eligible active duty servicewomen receiving long-acting reversible contraception with extended supply (Measure of Performance 3 eFigure 4. Monthly percentage of eligible active duty servicewomen with extended supply provided by permanent contraception (Measure of Performance 3) eFigure 5. Monthly percentage of eligible active duty servicewomen with short-acting reversible contraception with extended supply (Measure of Performance 3) eReferences

Supplement 2. Data Sharing Statement
