# Supplementary material for: Contraceptive Prescribing and Dispensing After the Defense Health Agency’s Policy Change
Source: JAMA Netw Open. 2025 Oct 27;8(10):e2539451. doi: 10.1001/jamanetworkopen.2025.39451 (PMC12559968; doi:10.1001/jamanetworkopen.2025.39451)
Supplement: Supplement 1. — eFigure 1. Department of Defense (DOD) contraceptive policy timeline eMethods 1. Identification and quantification of contraceptive methods eMethods 2. Measures of Performance (MOPs) Numerator and Denominator Definitions eFigure 2. Direct vs purchased care interrupted time series analysis eFigure 3. Monthly percentage of eligible active duty servicewomen receiving long-acting reversible contraception with extended supply (Measure of Performance 3 eFigure 4. Monthly percentage of eligible active duty servicewomen with extended supply provided by permanent contraception (Measure of Performance 3) eFigure 5. Monthly percentage of eligible active duty servicewomen with short-acting reversible contraception with extended supply (Measure of Performance 3) eReferences [file jamanetwopen-e2539451-s001.pdf]

Supplemental Online Content

Juneau R, K.C. G, Rittel AG, et al. Contraceptive prescribing after the defense health agency's policy change. *JAMA Netw Open.* 2025;8(10):e2539451.
doi:10.1001/jamanetworkopen.2025.39451

eFigure 1. Department of Defense (DOD) contraceptive policy timeline

eMethods 1. Identification and quantification of contraceptive methods

eMethods 2. Measures of Performance (MOPs) Numerator and Denominator Definitions

eFigure 2. Direct vs purchased care interrupted time series analysis

eFigure 3. Monthly percentage of eligible active duty service women receiving long-acting reversible contraception with extended supply (Measure of Performance 3)

eFigure 4. Monthly percentage of eligible active duty service women with extended supply provided by permanent contraception (Measure of Performance 3)

eFigure 5. Monthly percentage of eligible active duty service women with short-acting reversible contraception with extended supply (Measure of Performance 3)

eReferences

This supplemental material has been provided by the authors to give readers additional information about their work.

eFigure1: Department of Defense (DOD) contraceptive policy timeline

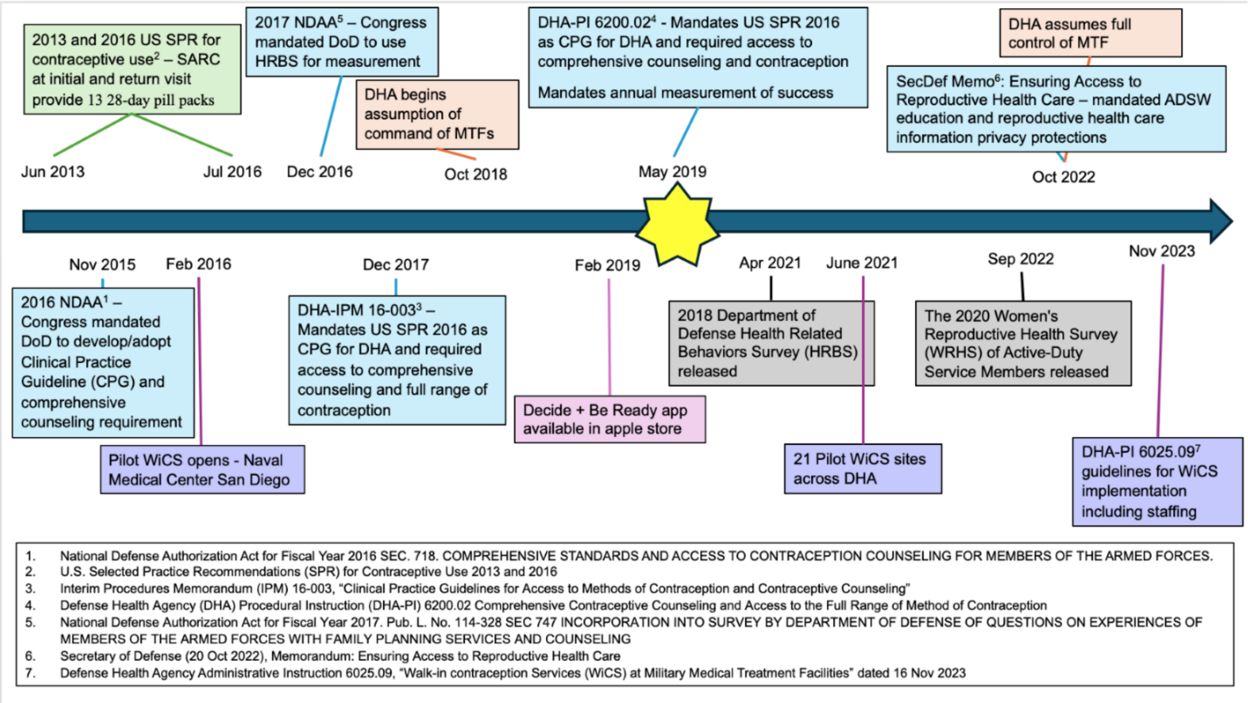

SPR: Selected Practice Recommendation; SARC: Short Acting Reversible Contraception; DoD: Department of Defense; NDAA: National Defense Authorization Act, WiCS: Walk-in Contraception Service

The Department of Defense (DoD) adopted the U.S. Selected Practice Recommendations (SPR) created by the Centers for Disease Control and Prevention in 2013 and updated in 2016 based on the World Health Organization's Selected Practice Recommendations of 2005.(updated in 2008). Congress mandated the Selected Practice Recommendations as part of Title 10[1] and the 2016 National Defense Authorization Act (NDAA).[2] A requirement for policy implementation metrics was later included as part of the 2017 NDAA.[3] The Defense Health Agency guided implementation of the U.S. Selected Practice Recommendations through the 2016-2017 Defense Health Agency Interim Procedure Memorandum (DHA-IPM) 16-003 Clinical Practice Guidelines for Access to Methods of Contraception and Contraceptive Counseling that mandated access to comprehensive counseling and a full range of contraception.[4,5]

© 2025 Juneau R et al. *JAMA Network Open*

eMethods1: Identification and quantification of contraceptive methods

Short-Acting Reversible Contraceptive (SARC)

For oral contraceptive medications, the quantity recorded in the claim was used to determine the days supply, except for products with a package size of 21 pills or a quantity of 1 to 14 pills. Four National Drug Codes identified with a package size of 21, the days supply was calculated by multiplying the quantity by 1.33 days to account for the absence of placebo pills. For prescriptions with quantities ranging from 1 to 14 pills, the reported quantity was multiplied by 28 days. For patches, the days supply was calculated by multiplying the quantity by 9.33 days. For vaginal rings (excluding Annovera, which equals 364 days), the days supply was calculated by multiplying the quantity by 28 days. Each injectable dispensed equaled a 90-day supply. Calculated days supplies were rounded to the nearest full month. Days supplies were capped as follows: injections (360 days, 4 injections), patches (378 days, 42 patches or 14 packages), rings (378 days, 14 rings), and oral medications (392 days, 14 packages).

Long Acting Reversible Contraceptives (LARCs)

International Classification of Diseases, Ninth Revision, Clinical Modification (ICD-9-CM) and International Classification of Diseases, Tenth Revision, Clinical Modification (ICD-10-CM) diagnosis codes, PDTS prescription data, Current Procedural Terminology (CPT) procedure codes, and Healthcare Common Procedure Coding System (HCPCS) codes were used to identify LARC and determine the months of supply. For an intrauterine device (IUD) with 36 months supply and placement on March 15, 2016, the first month of supply started on March 1, 2016, and the last month of supply ended on February 28, 2019. If CPT or ICD codes for a LARC were identified and the specific date of implantation or product receipt could not be identified, the

© 2025 Juneau R et al. *JAMA Network Open*

following supplys were used: implants provide 18 months of supply and IUD provide 5 years of supply. If the LARC was removed, supply continues through the month prior to the index month. For example, if the LARC is removed on November 5, 2018, the supply would be from March 1, 2016, until October 31, 2018. This variation in the lookback period is to ensure those who are still covered from their LARC device prior to study period are labeled as having supply during our study period.

Permanent contraception

Permanent contraception procedure codes (e.g., Z98.51, Z30.2) were identified using ICD-9-CM, ICD-10-CM, and CPT codes. For permanent contraception reversal procedures, the index month of reversal is not included as a month of supply, similar to LARCs. For a permanent contraception reversal procedure on November 15, supply ended on October 31.

No Contraception

The no contraception group included active duty service women (ADSW) who had no contraception supply (SARC, LARC placement, or permanent contraception) or during the entire study period.

eMethods 2: Measures of Performance (MOPs) Numerator and Denominator Definitions

MOP 1 – Monthly Percent of Eligible active duty service women (ADSW) with Extended SARC Days Supply: The numerator is the number of eligible ADSW with an active extended SARC prescription supply (≥168 days supply in a single dispense event) during the reported month. The denominator is the number of eligible ADSW with an active SARC prescription for the reported month.

© 2025 Juneau R et al. *JAMA Network Open*

MOP 2 – Monthly Percent of SARC Contraceptive Prescriptions Dispensed for an Extended Days Supply: The numerator is the number of extended duration SARC prescriptions dispensed to eligible ADSW in the reported month. The denominator is the total number of SARC prescriptions dispensed to eligible ADSW during the reported month.

MOP 3 – Monthly Percent of Eligible ADSW with Any Extended Supply Contraception (SARC, LARC, or Permanent contraception) The numerator is the number of eligible ADSW with extended contraceptive supply for the reported month. The denominator is the total number of eligible ADSW in the reported month.

MOP 4 – Monthly Percent of Prescribers Providing Eligible ADSW with Extended Contraceptive Supply: The numerator is the number of prescribers who wrote at least one SARC prescription for ≥168 days supply, placed a LARC, or performed a permanent contraception procedure (extended contraceptive supply) in the reported month. The denominator is the number of prescribers who wrote at least one SARC prescription, placed a LARC, or performed a permanent contraception procedure for an eligible ADSW in the reported month.

MOP 5 – Monthly Percent of ADSW with Contraceptive Supply with Extended Supply Contraception (SARC, LARC, or Permanent contraception): The numerator is the number of ADSW with extended contraceptive supply for the reported month. The denominator is the total number of eligible ADSW who received any form of contraceptive in the reported month.

© 2025 Juneau R et al. *JAMA Network Open*

eFigure2: Direct vs purchased care interrupted time series analysis

<table>
<tr><td colspan="2">A. Monthly Percent of Eligible ADSW with Extended Short-Acting Reversible Contraception (SARC) Days Supply (MOP1)</td></tr>
<tr><td>Direct Care</td><td>Purchased Care</td></tr>
<tr><td>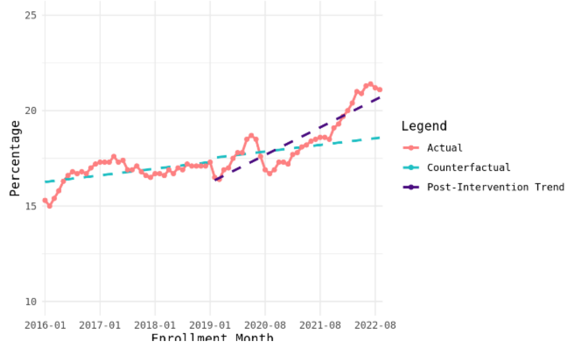
</td><td>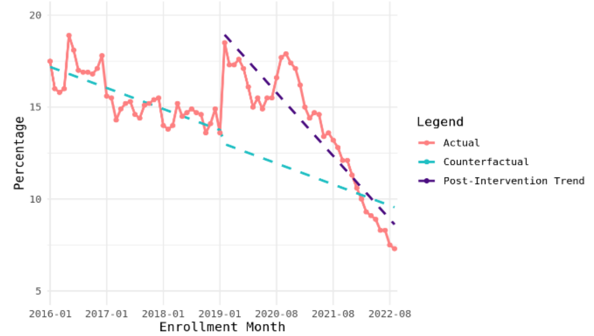
</td></tr>
<tr><td colspan="2">B. Monthly Percent of SARC Contraceptive Prescriptions Dispensed for an Extended Days Supply (MOP 2)</td></tr>
<tr><td>Direct Care</td><td>Purchased Care</td></tr>
<tr><td>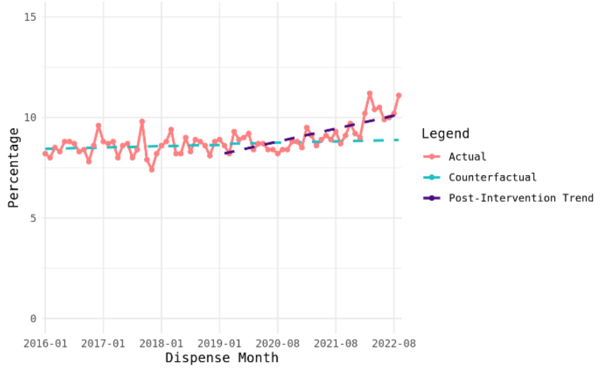
</td><td>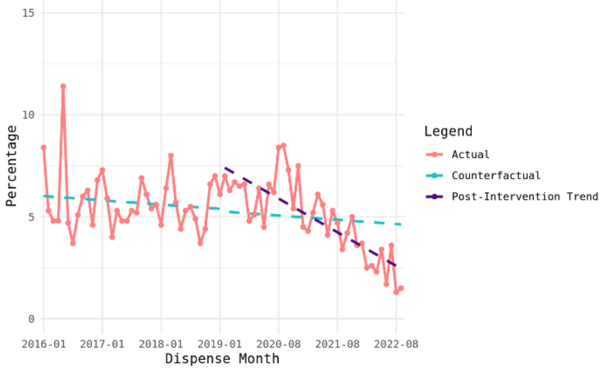
</td></tr>
<tr><td colspan="2">C. Monthly Percent of Eligible ADSW with Any Extended Supply Contraception (SARC, LARC, or Permanent Contraception) (MOP3)</td></tr>
<tr><td>Direct Care</td><td>Purchased Care</td></tr>
</table>

© 2025 Juneau R et al. *JAMA Network Open*

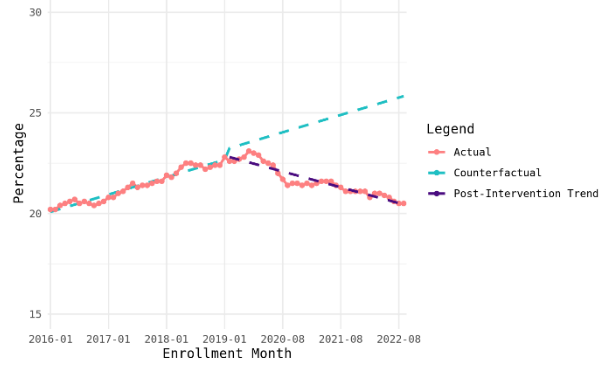

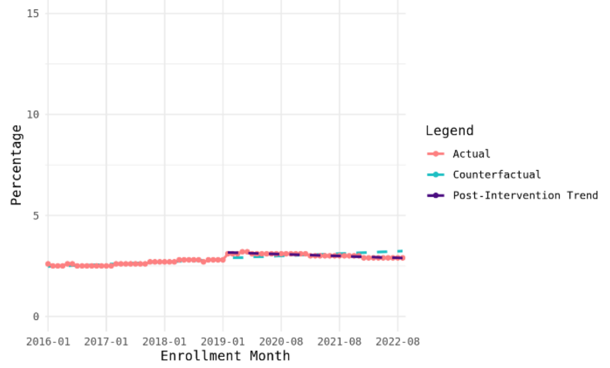

D. Monthly Percent of Prescribers Providing Eligible ADSW with Extended Contraceptive Supply (MOP4)

Direct Care	Purchased Care

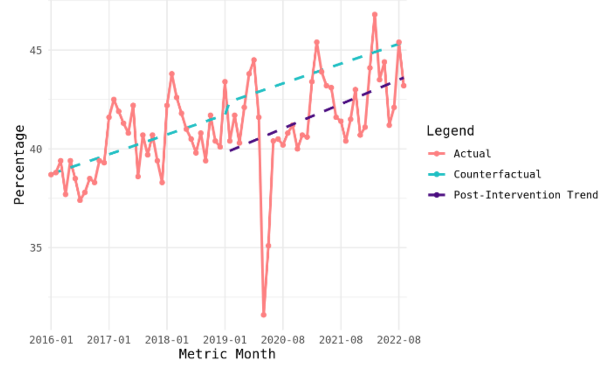

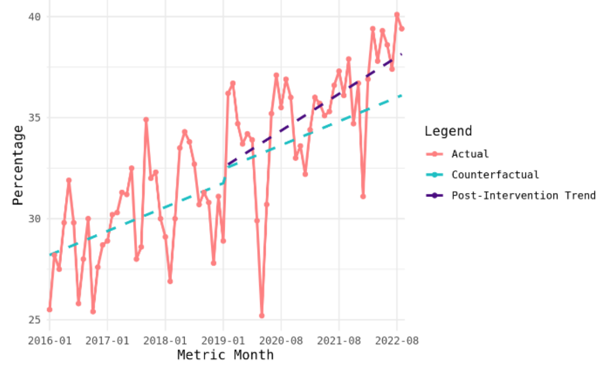

E. Monthly Percent of ADSW with Contraceptive Supply Any Extended Supply Contraception (SARC, LARC, or Permanent Contraception Procedures) (MOP5)

Direct Care	Purchased Care

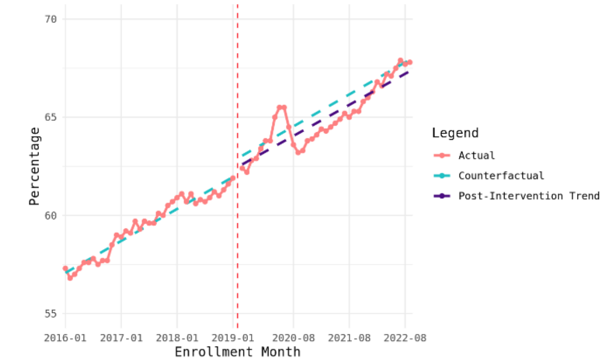

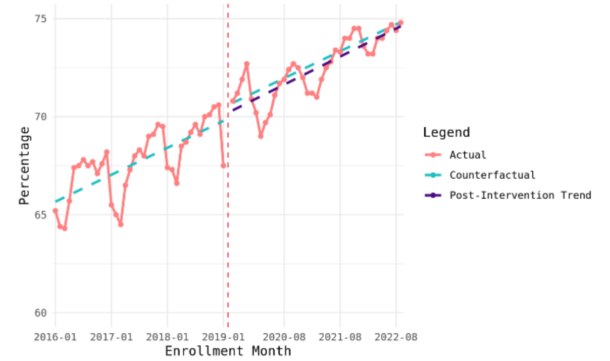

© 2025 Juneau R et al. *JAMA Network Open*

eFigure3: Monthly percentage of eligible active duty service women receiving long-acting reversible contraception with extended supply (Measure of Performance 3)

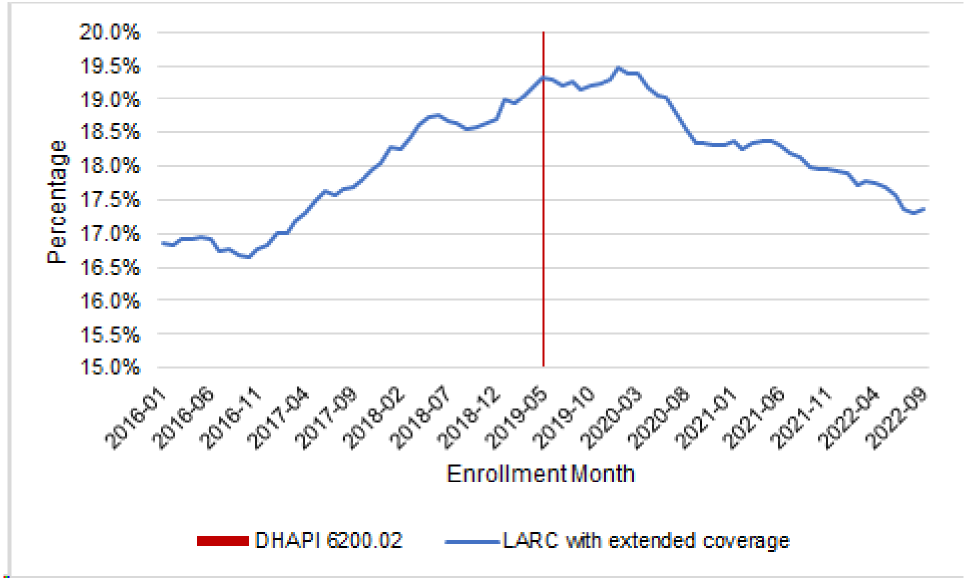

© 2025 Juneau R et al. *JAMA Network Open*

eFigure4: Monthly percentage of eligible active duty service women with extended supply provided by permanent contraception (Measure of Performance 3)

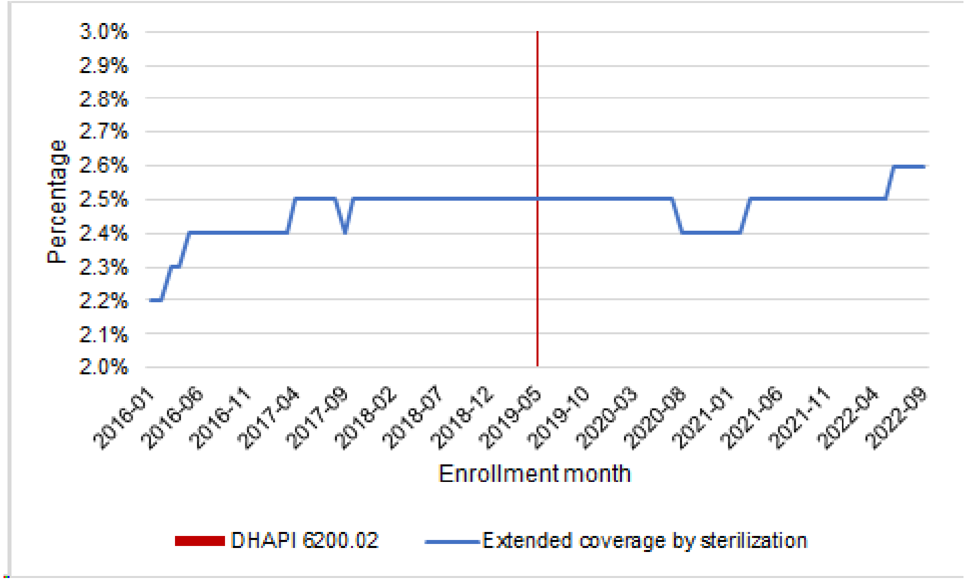

© 2025 Juneau R et al. *JAMA Network Open*

eFigure5: Monthly percentage of eligible active duty service women with short-acting reversible contraception with extended supply (Measure of Performance 3)

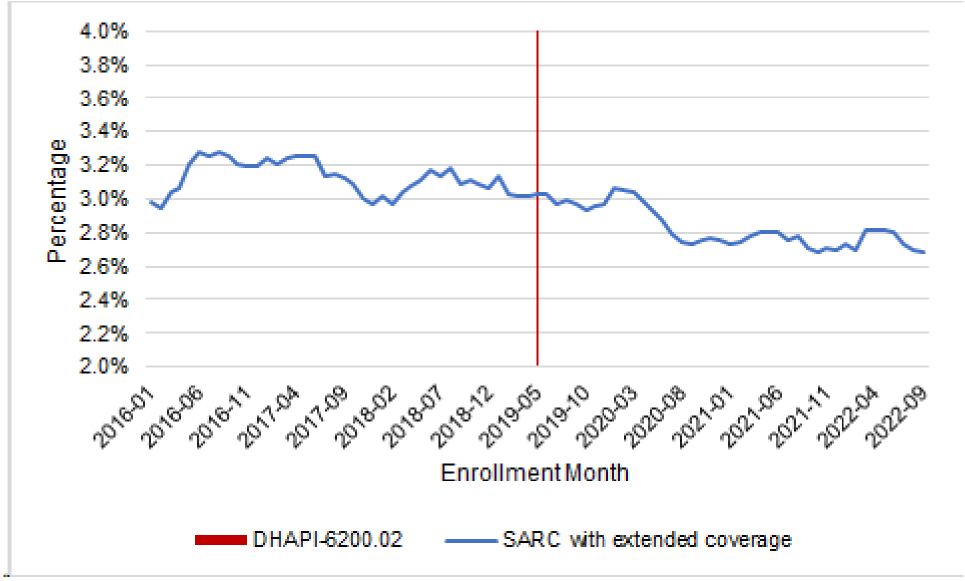

© 2025 Juneau R et al. *JAMA Network Open*

eReferences

1. 10 U.S.C. § 1074d - U.S. Code Title 10. Armed Forces § 1074d | FindLaw. FindLAW. Accessed September 11, 2024. https://codes.findlaw.com/us/title-10-armed-forces/10-usc-sect-1074d/

2. *National Defence Authorization Act For Fiscal Year 2016*.; 2015:Pub. L. No. 114-92 SEC 718 (2015) 129 STAT. 869.

3. *National Defence Authorization Act For Fiscal Year 2017*.; 2016. https://www.congress.gov/114/plaws/publ328/PLAW-114publ328.pdf

4. Curtis KM. U.S. Selected Practice Recommendations for Contraceptive Use, 2016. *MMWR Recomm Rep*. 2016;65. doi:10.15585/mmwr.rr6504a1

5. *Defence Health Agency (DHA) Interim Procedures Memorandum (IPM) 16-003, "Clinical Practice Guidelines for Access to Methods of Contraception and Contraceptive Counseling."* Department of Defence; 2017.
