# Supplementary material for: Contraceptive Prescribing and Dispensing After the Defense Health Agency’s Policy Change
Source: JAMA Netw Open. 2025 Oct 27;8(10):e2539451. doi: 10.1001/jamanetworkopen.2025.39451 (PMC12559968; doi:10.1001/jamanetworkopen.2025.39451)
Supplement: Supplement 2. — Data Sharing Statement [file jamanetwopen-e2539451-s002.pdf]

Data Sharing Statement

Juneau. Contraceptive Prescribing and Dispensing After the Defense Health Agency's Policy Change. *JAMA Netw Open*. Published October 27, 2025.
doi:10.1001/jamanetworkopen.2025.39451

Data
Data available: No

Additional Information
Explanation for why data not available: The data is deidentified and on a secure server owned by the Defense Health Agency.
